# Microstructure, Non-Basal Texture and Strength-Ductility of Extruded Mg–6Bi–3Zn Alloy

**DOI:** 10.3390/ma17153835

**Published:** 2024-08-02

**Authors:** Xin Li, Jian Mao, Xuefei Huang, Weigang Huang

**Affiliations:** College of Material Science and Engineering, Sichuan University, Chengdu 610065, China

**Keywords:** microstructure, texture, dynamic recrystallization, mechanical property, Mg–Bi–Zn alloy

## Abstract

To investigate the influence of Zn-alloying on the microstructure and tensile mechanical properties of Mg–6Bi alloy after hot extrusion, a new ternary Mg–6Bi–3Zn alloy was prepared by extrusion at 300 °C. The microstructures, texture, dynamic precipitates and tensile mechanical behaviors of the extruded alloy were characterized by transmission electron microscopy (TEM), X-ray diffraction (XRD), electron backscattered diffraction (EBSD) and a material testing machine at room temperature. After extrusion, the Mg–6Bi–3Zn alloy possesses a bimodal microstructure with elongated large unrecrystallized (unDRXed) grains and fine dynamic recrystallized (DRXed) grains. In addition, non-basal <20$\bar{2}$1>//ED, <44$\bar{8}$3>//ED and <11$\bar{2}$1>//ED textures are observed within DRXed grains due to the Zn addition, leading to texture weakening in the extruded Mg–6Bi–3Zn alloy. Zn addition facilitates the dynamic precipitation behavior, leading to a 12.2% area fraction of Mg_3_Bi_2_ precipitates with an average size of 39.2 nm. Furthermore, incorporation of Zn atoms in Mg_3_Bi_2_ phases and segregation of Zn at the grain boundary are found. The extruded Mg–6Bi–3Zn alloy exhibits a tensile strength of 336 ± 7.1 MPa and a yield strength of 290 ± 5.5 MPa, as well as an elongation of 11.5%. Therefore, Zn addition is beneficial to enhance strength and keep good ductility for the extruded Mg–6Bi–3Zn alloy.

## 1. Introduction

Mg-based alloys with high strength and good ductility are important for industrial applications [1]. However, at room temperature, the poor formability or ductility of magnesium alloys do not adequately satisfy many engineering applications. Previous literature [1,2,3,4,5,6] revealed that Mg alloys added with rare earth (RE) can weaken the basal texture and promote non-basal slip due to the formation of RE texture. In addition, Ca and Zn, as non-rare earth elements, also play an effective role in reducing the basal texture intensity and activating prismatic and pyramidal slip systems, leading to improved ductility [7,8,9,10]. The role of these elements in improving ductility can be mainly ascribed to decreasing the stacking fault energy (SFE) of the Mg matrix, hindering the movement of the grain boundary (GB) and dislocations by the solute drag effect and promoting a non-basal slip of dislocations [3,6,7,8,9,10,11].

Nowadays, Mg–Bi series alloys with other elements have been investigated owing to their high strength and excellent extrudability. To enhance strength, Al, Sn, Ca and Mn elements were individually incorporated into Mg–Bi alloy, resulting in high strength [12,13,14,15,16,17]. The research from Go et al. [12] demonstrates that the Mg–5Bi–3Al alloy can be extruded with 67 m/min extrusion speed at 400 °C due to the presence of the Mg_3_Bi_2_ phase with very good thermal stability, exhibiting excellent extrudability. However, the strength of the alloy is decreased by extrusion at a high temperature of 400 °C. Mg–Bi–Ca alloy reveals good ductility, which is due to Ca addition resulting in texture weakening and fine grain size [14,15]. Furthermore, Mg–8Bi–1Al–1Zn, Mg–6Bi–3Al–1Zn, Mg–Bi–Mn–Zn and Mg–5Bi–5Sn–1Mn alloys with multi-alloying elements demonstrate high strength and good ductility [18,19,20,21].

As far as we know, besides the closely researched Mg–6Bi–3Al–1Zn alloy with high Al and low Zn content, the ternary Mg–6Bi–3Zn alloy has not been investigated. Accordingly, a new ternary Mg–6Bi–3Zn alloy was prepared by extrusion at 300 °C in this study. Then, the characteristics of the microstructure, including dynamic recrystallization (DRX), texture, dynamic precipitates and tensile mechanical properties, were studied. This research aims to develop a Mg–6Bi–3Zn alloy with high strength and good ductility by means of Zn promoting dynamic precipitation and weakening basal texture in Mg–Bi alloy during extrusion.

## 2. Materials and Methods

The experimental Mg–6Bi–3Zn (wt.%) alloy was made using an electric resistance furnace under a protective atmosphere with CO_2_ (99%) and SF_6_ (1%) gases. The pure metals used for producing experimental alloy are Mg, Bi and Zn with a purity of 99.9%. The real chemical compositions of experimental Mg–6Bi–3Zn alloy are 5.92 wt.% Bi, 3.01 wt.% Zn and a balance of Mg, which were measured by an inductively coupled plasma optical emission spectroscope (ICP, Agilent 5100, Santa Clara, CA, USA). Before extrusion, all cast samples, covered by graphite powder, were solid-solution heat-treated at 450 °C for 10 h, then at 510 °C for 2 h after that, followed by water quenching. Subsequently, the solution-treated samples were extruded at 300 °C with a extrusion ratio of 25:1 under a die-exit speed of 2 m/min. After extrusion, bars with 5 mm diameter were obtained. The microstructure characteristics of the extruded Mg–6Bi–3Zn alloy were examined using X-ray diffraction (XRD, Smart Lab, Rigaku, Tokyo, Japan), transmission electron microscopy (TEM, FEI Titan Cubed Themis G2 300, Ames Laboratory, Ames, IA, USA), equipped with a Super-X EDS system and Cs probe corrector, and electron backscattered diffraction (EBSD, JSM-7200F, DEAX Velocity^TM^ Super, Wolfforth, TX, USA). The corresponding data obtained from EBSD were analyzed with the AZtecCrystal software version 2.1 (Oxford Instruments, Abingdon, UK). The contents and size of precipitates in alloy were analyzed with Image-Pro Plus 6.0 software after extrusion.

The samples for the tensile test were cut from the extruded bars along the extrusion direction (ED), which has a gauge size of ϕ2.5 mm × 25 mm. The tensile test was performed using a tensile testing machine (DDL-100) at room temperature and a strain rate of 1 × 10^−3^ s^−1^.

## 3. Results and Discussion

### 3.1. EBSD Microstructure

**Figure 1:**
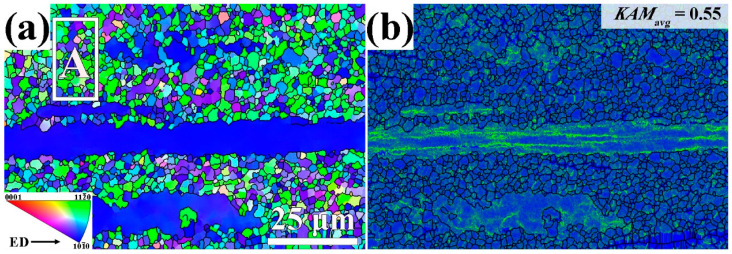
The microstructure of the extruded Mg–6Bi–3Zn alloy: (**a**) IPF map, (**b**) KAM map.

Figure 1a illustrates the inverse pole figure (IPF) image of the Mg–6Bi–3Zn alloy that was extruded at 300 °C. After extrusion, the Mg–6Bi–3Zn alloy demonstrates a bimodal microstructure, which consists of large elongated unrecrystallized (unDRXed) grains along the ED and fine dynamic recrystallized (DRXed) grains. As indicated in Figure 1a, the elongated unDRXed grains show the <10$\bar{1}$0> crystallographic orientation, which is parallel to the ED. Although the DRXed grains do not exhibit an obviously preferred orientation, most of the DRXed grains show close to <11$\bar{2}$0> crystallographic orientation. It is determined from Figure 1a that the average size and area fraction of fine DRXed grains is 3.3 μm and 75.0%, respectively. The average size of unDRXed grains is 30.1 μm. According to the previous work [22], the Mg–6Bi alloy extruded at the same extrusion parameters reveals 64.1% of the area fraction of the DRXed grains. It illustrates that Zn in Mg–6Bi alloy can effectively promote DRX, which results in the increase in content of the DRXed grains.

Zn promoting DRX and increasing the fraction of DRX can be attributed to two main aspects. One aspect is the presence of a high content of dynamic precipitates in Mg–6Bi–3Zn alloy due to Zn promoting dynamic precipitation behavior. The size of the dynamic precipitates is 39.2 nm, as seen in Section 3.3. It was indicated by Shen et al. [23] and Zou et al. [24] that fine precipitates with nanosize can obviously promote the continuous dynamic recrystallization (CDRX). The nanosized precipitates can impede dislocation movement by the pinning effect, resulting in the enhancement of dislocation density. Consequently, this increases the driving force of CDRX [23]. Another aspect is that Zn can decrease the SFE in Mg alloys [25]. Low SFE can make it difficult for dislocation movements by cross-slip and climb slip, which hinders the occurrence of dynamic recovery (DRV). As a result, the retained high density of dislocations can facilitate the formation of new grains by discontinuous dynamic recrystallization (DDRX).

The KAM (kernel average misorientation) map in Figure 1b indicates that a high density of dislocations is distributed near the high angle grain boundaries (HAGBs) and within unDRXed grains. Some dislocations in the DRXed grains are near the HAGBs between the DRXed grains. This means that highly accumulated dislocations can bring about a stress concentration at the interface of these HAGBs for both DRXed and unDRXed grains.

### 3.2. Texture Characteristics of the Extruded Alloy

**Figure 2:**
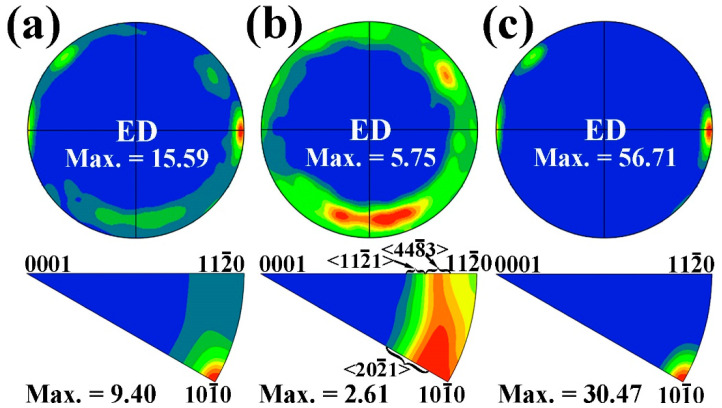
(0001) pole figures and ED IPF_S_ of extruded Mg–6Bi–3Zn alloy: (**a**) entire regions, (**b**) DRXed regions, and (**c**) unDRXed regions.

Figure 2 demonstrates the (0001) pole figures and ED inverse pole figures (ED IPF_S_) in the extruded Mg–6Bi–3Zn alloy. As seen from the (0001) pole figure and the ED IPF in the entire region indicated in Figure 2a, it is clearly revealed that the c-axes of a large amount of grains are perpendicular to the ED, and the <10$\bar{1}$0> crystal directions are obviously parallel to the ED, which indicates the presence of a strong (0001)//ED, <10$\bar{1}$0>//ED basal texture. It is seen from the pole figure and the ED IPF that the maximum intensity of th texture is 15.59 and 9.40 for the extruded Mg–6Bi–3Zn alloy, respectively. Beside the strong <10$\bar{1}$0>//ED basal texture, some weak <11$\bar{2}$0>//ED textures are also observed. As illustrated in Figure 2c, the large unDRXed grains only exhibit a strong <10$\bar{1}$0>//ED basal texture. However, Figure 2b clearly shows that the c-axes of most DRXed grains in the extruded alloy is inclined by 74.39° away from the ED, which means that the (0001) plane of a large number of DRXed grains is nearly parallel to the ED. Furthermore, the ED IPF in Figure 2b reveals the existence of the <20$\bar{2}$1>//ED, <44$\bar{8}$3>//ED and <11$\bar{2}$1>//ED non-basal textures with relatively lower intensity than that of the <10$\bar{1}$0>//ED basal texture. Among them, the <11$\bar{2}$1>//ED texture is regarded as a RE texture [3,26]. Owing to the deviation angle of about 6.3° between <44$\bar{8}$3> and <11$\bar{2}$1>, it is reasonable to refer to <44$\bar{8}$3>//ED as the RE texture. Therefore, it can be clearly defined that the formation of these non-basal textures indicated in Figure 2b for Mg–6Bi–3Zn alloy is ascribed to the Zn addition in Mg–6Bi alloy.

**Figure 3:**
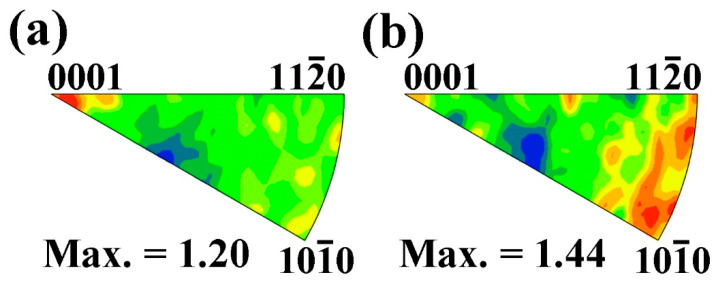
The IGMA distribution maps of extruded Mg–6Bi–3Zn alloy: (**a**) entire DRXed regions and (**b**) DRXed region A.

To further discuss the texture development of the extruded Mg–6Bi–3Zn alloy, the in-grain misorientation axes (IGMA) analysis was used with EBSD measurement. IGMA analysis is based on the main assumption that the lattice rotation about a crystallographic axis arises from the activation of the slip system [27,28]. The rotation axis is perpendicular to both the slip direction and the slip plane normal direction. This rotation axis is termed the “Taylor axis”, which corresponds to different slip systems, as shown in Table 1. From the present EBSD measurement, the IGMA distribution for the entire DRXed grain region is given in Figure 3a. It clearly indicates that the IGMA is distributed intensively toward the <0001> rotation axis. Furthermore, it can be found that the distribution of IGMA spreads slightly away from the <0001> direction, which can reduce the maximum intensity of the <0001> rotation. The result in Figure 3a can suggest that the prismatic <a> slip of dislocations can be activated during the extrusion deformation due to the rotation of th lattice about the <0001> axis, as indicated in Table 1. As a result, during extrusion, the activated prismatic <a> slip system in the grains can cause the <10$\bar{1}$0> crystallographic direction to align with the extrusion direction, leading to the formation of a <10$\bar{1}$0>//ED basal texture [29]. However, the spread of IGMA distribution from the <0001> rotation axis can suggest the possible other slip system activations, such as pyramidal <c + a> slip systems, which can cause the lattice to rotate about other Taylor rotation axes [27], resulting in the reduction of the IGMA distribution intensity of the <0001> rotation axis. Therefore, it can be suggested that the <10$\bar{1}$0>//ED basal texture can be spread due to the deviation of the <0001> rotation axis, which reduces the intensity of the <10$\bar{1}$0>//ED basal texture. Besides the concentrated <0001> axis, a weak <10$\bar{1}$0> rotation axis distribution can be found in Figure 3a, which corresponds to the activation of both the basal slip system and pyramidal II slip system due to two slip systems having the same <10$\bar{1}$0> rotation axis. To further reveal the characteristic of these dislocations, the DRXed grain region A indicated in Figure 1a is selected. From the IGMA distribution map of the selected DRXed grain region presented in Figure 3b, it can be found that the distribution of the lattice rotation axis is deviated from the <10$\bar{1}$0> axis and spread toward to the <11$\bar{2}$0>//ED direction, which should arise from the basal <a> slip and pyramidal II <c + a> slip of dislocations, as well as other non-basal slips. These activated, other non-basal slip systems give rise to the lattice rotation axis corresponding to the <u v t w> direction. In the present work, the RE textures of <11$\bar{2}$1>//ED and <44$\bar{8}$3>//ED that occurred in Mg–6Bi–3Zn alloy during extrusion coincides with the results reported by Imandoust et al. [30] in Mg–RE alloy. This type of RE texture results from the fact that the recovery of high density basal/<c + a> dislocations induces the “backward rotation” of recrystallized grains during recrystallization toward the [0001] pole.

Based on the above discussion, it can be understood that the occurrence of the non-basal texture closely relates to the non-basal dislocation slip, including the prismatic <a> and pyramidal <c + a> dislocations. Hase et al. [31] indicated that the Zn element in Mg alloy can activate the non-basal slip of dislocations, especially for prismatic dislocation slip, due to the reduction of the SFE of the prismatic dislocation slip. The decrease in SFE can cause the cross-slip of basal dislocations to be difficult, resulting in the reduction of the probability of basal dislocation movements. In contrast, it promotes the formation of non-basal dislocations. As indicated by Sandlöbes et al. [32], the I_1_ stacking faut can act as the nucleation site of <c + a> non-basal dislocations due to the I_1_ stacking fault integrating with pyramidal partial dislocations. Therefore, low SFE can facilitate the formation of non-basal dislocation slips.

Furthermore, Zn addition can reduce the critical resolved shear stress (CRSS) of the prismatic dislocation slip [33]. Stanford et al. [8] indicated that the CRSS ratio between the prismatic slip and basal slip of dislocations can be reduced by the addition of Zn. Jang et al. [34] indicated that Zn addition in Mg alloy can increase the binding energy between the basal dislocations and Zn solutes, resulting in the increase of the CRSS of basal slips. Although Zn also increases the CRSS of pyramidal slip of dislocations due to the increase in binding energy, the increment of CRSS is smaller than that for basal slips. Therefore, Zn addition can reduce the difference in CRSS between the various slip systems, resulting in promoting the activation of <c + a> slip systems.

In addition, the solute atoms segregated at the grain boundaries and dislocations demonstrate a significant impact on texture weakening, which can impede grain boundary migration and basal slip of dislocations, resulting in promoting non-basal slip of dislocations [3]. In addition, when the solute segregation at the grain boundary hinders the migration of the grain boundary by solute drag, the DDRX behavior can be retarded. Thus, the DRX mechanism changes from DDRX to CDRX by promoting the formation and rotation of sub-grains. The occurrence of CDRX behavior can bring about the recrystallization texture or non-basal texture, which results in the weakening of the strong basal texture [35]. The evidence of Zn atom segregation at the grain boundary in the present experiment is illustrated in the next section.

### 3.3. Dynamic Precipitation Behavior

**Figure 4:**
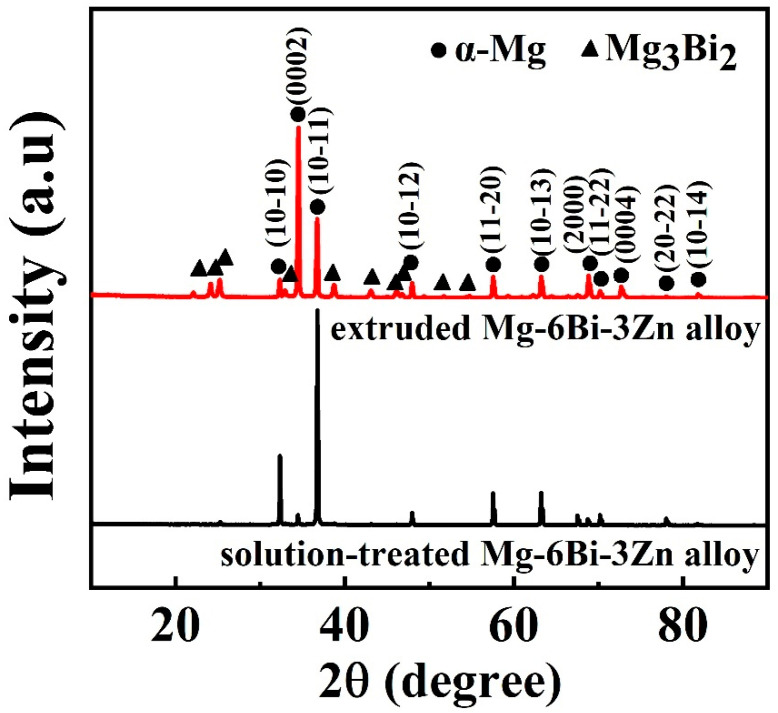
XRD patterns of Mg–6Bi–3Zn alloys treated by solid-solution treatment and extrusion.

Figure 4 presents the XRD patterns of the Mg–6Bi–3Zn alloy treated by solution treatment and extrusion. It can be seen from Figure 4 that both solution-treated and extruded alloys exhibit the α-Mg and Mg_3_Bi_2_ phases. However, the diffraction peak intensity of the Mg_3_Bi_2_ phase in the extruded alloy is greater than that in the solution-treated alloy, which means that a large number of Mg_3_Bi_2_ phases formed in the extruded alloy due to the occurrence of dynamic precipitation behavior during extrusion at 300 °C. The dislocation density in the alloy can be increased during hot extrusion deformation. The high dislocation density can provide diffusion channels for solutes and nucleation sites for precipitates, thus promoting the content of the precipitates [36]. Moreover, the (0002) diffraction peak intensity of the α-Mg phase in the extruded alloy is significantly higher than that in the solution-treated alloy. This indicates that the extruded alloy exhibits a strong basal texture, as demonstrated in Section 3.2.

**Figure 5:**
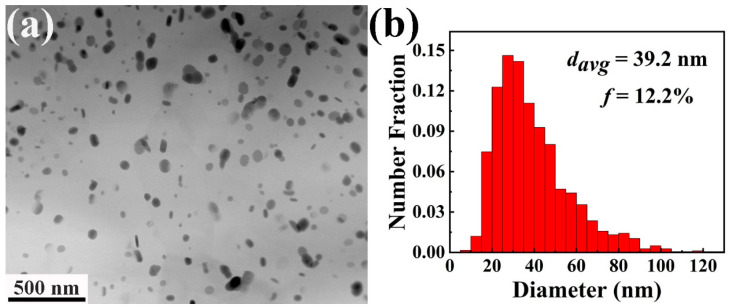
The TEM image and statistical histogram of the diameters of the dynamic precipitates of Mg_3_Bi_2_ in the extruded Mg–6Bi–3Zn alloy. (**a**) Bright-field TEM image, (**b**) statistical histogram of the diameters of the dynamic precipitates.

Figure 5 demonstrates the TEM image and statistical histogram of the diameters of the dynamic precipitates. The TEM image indicates that most of the precipitates show an equiaxed morphology and are distributed uniformly in the α-Mg matrix. The average particle size and area fraction of the precipitates, determined using four TEM images, are 39.2 nm and 12.2%, respectively, as indicated in Figure 5b. According to the XRD results, these dynamic precipitates should correspond to the Mg_3_Bi_2_ phase.

**Figure 6:**
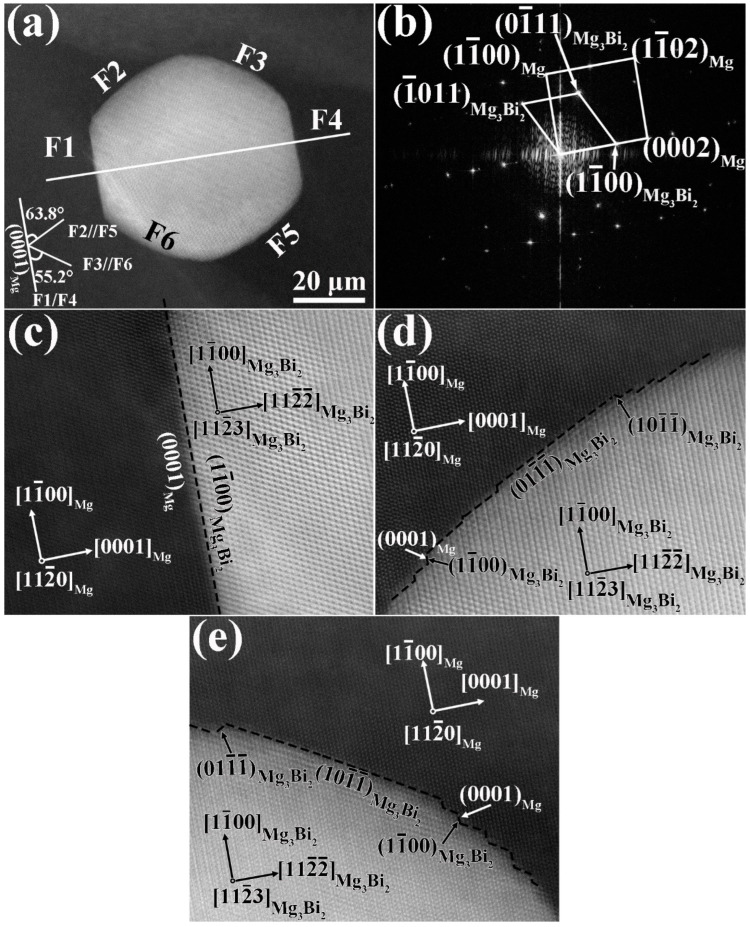
The HAADF-STEM images of a dynamic Mg_3_Bi_2_ precipitate: (**a**) the image of the precipitate, (**b**) the electron diffraction pattern from FFT, (**c**) the image of F1 facet, (**d**) the image of the F2 facet, and (**e**) the image of the F3 facet.

Figure 6 presents the high-angle angular dark field-scanning transmission electron microscopy (HAADF-STEM) images of a dynamic precipitate exhibiting a hexagonal shape and the interface feature between the precipitate and the α-Mg matrix. The precipitate was indexed using the fast Fourier transform (FFT) pattern to be the Mg_3_Bi_2_ phase. The orientation relationships (ORs) between the α-Mg matrix and the Mg_3_Bi_2_ phase are obtained as follows: [11$\bar{2}$3]_Mg3Bi2_//[11$\bar{2}$0]_Mg_, (1$\bar{1}$00)_Mg3Bi2_//(0001)_Mg_ and (11$\bar{2}$$\bar{2}$)_Mg3Bi2_//(1$\bar{1}$00)_Mg_. To understand the interfacial crystallographic characteristics between the α-Mg matrix and the Mg_3_Bi_2_ phase, each facet of the Mg_3_Bi_2_ phase is marked as F1–F6, where the F2–F5, F1–F4 and F3–F6 facets are parallel to each other. A pair of parallel facets should be equivalent crystallographic facets, and the angles between the (0001)_Mg_ trace and these facets are indicated in Figure 6a, wherein the F1–F4 facets are parallel to the (0001)_Mg_ trace. Figure 6c–e illustrates the interface characteristic between the F1, F2, F3 facets and the α-Mg matrix, respectively. From Figure 6c, it is found that the F1 facet has a flat broad interface, which exhibits (1$\bar{1}$00)_Mg3Bi2_//(0001)_Mg_ OR, and the interface trace is along (1$\bar{1}$00)_Mg3Bi2_. Therefore, this kind of interface, such as F1, belongs to the atomical flat interface. However, it is observed from Figure 6d,e that the F2 and F3 facets of the Mg_3_Bi_2_ phase consist of short flat facets and some terraces. The short flat facets of F2 and F3 correspond to (01$\bar{1}$$\bar{1}$)_Mg3Bi2_ and (10$\bar{1}$$\bar{1}$)_Mg3Bi2_, respectively. By analysis, it is found that the (01$\bar{1}$$\bar{1}$)_Mg3Bi2_ and (10$\bar{1}$$\bar{1}$)_Mg3Bi2_ planes exhibit a deviation angle of 1.95° from the (1$\bar{1}$01)_Mg_ and (10$\bar{1}$$\bar{1}$)_Mg3Bi2_ planes, respectively. This means that the interface between the short flat facet of the Mg_3_Bi_2_ phase and the plane of the α-Mg matrix reveals no strict crystallographic relationship. As exhibited in Figure 6d,e, many steps form on the interface of the F2 and F3 facets due to the reduction of interfacial energy. Two types of steps in the interface can be seen in Figure 6d,e. One step indicates a (1$\bar{1}$00)_Mg3Bi2_//(0001)_Mg_ crystallographic relationship. The other types have no crystallographic relationship between the interface of the Mg_3_Bi_2_ phase and the α-Mg matrix, including steps with (10$\bar{1}$$\bar{1}$)_Mg3Bi2_ and steps with (01$\bar{1}$$\bar{1}$)_Mg3Bi2_.

**Figure 7:**
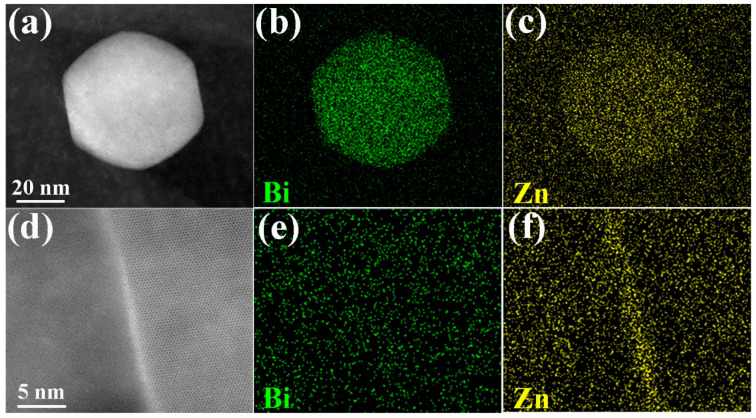
EDS mapping results of the dynamic precipitate in Figure 6a and grain boundary: (**a**–**c**) the dynamic precipitate and (**d**–**f**) the grain boundary.

Figure 7 demonstrates the EDS mappings of the dynamic precipitate and a grain boundary in the extruded Mg–6Bi–3Zn alloy. The result reveals that Zn atoms are distributed within the Mg_3_Bi_2_ phase, as demonstrated in Figure 7c. Wang et al. [19] indicate that the Zn atoms dissolving into the Mg_3_Bi_2_ phase should be energetically favorable according to the results of thermodynamic calculation, resulting in increasing the stability of the Mg_3_Bi_2_ phase. As a result, it can accelerate the dynamic precipitation, leading to an increase in the content of Mg_3_Bi_2_ precipitates and the refinement of dynamic precipitate size. It should be noted that only Zn atoms are segregated at the grain boundary in the extruded Mg–6Bi–3Zn alloy, as exhibited in Figure 7f. The atomic radius (0.134 nm) of a Zn atom is smaller than that of Mg (0.160 nm) [37]. Therefore, Zn atoms prefer to segregate at compressed regions of grain boundary dislocations, aiming to reduce the elastic strain energy of dislocations and grain boundary energy [19,38]. In addition, the segregation of Zn atoms at the grain boundary can impede grain boundary migration during hot extrusion, which results in the refinement of DRXed grains and texture weakening due to the solute drag effect [3].

### 3.4. Strength and Ductility

**Figure 8:**
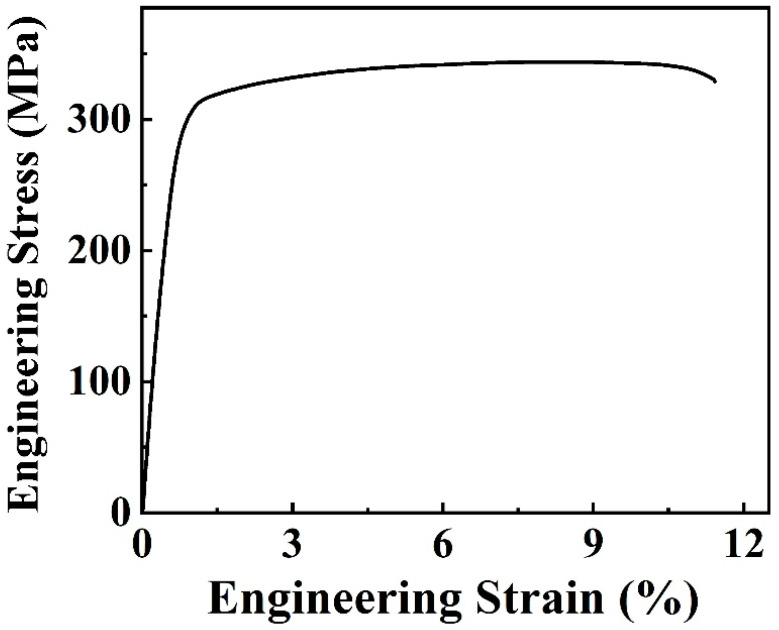
The engineering tensile stress–strain curve of the extruded Mg–6Bi–3Zn alloy.

Figure 8 exhibits the engineering tensile stress–strain curve of the extruded Mg–6Bi–3Zn alloy, and the relevant mechanical properties are listed in Table 2. For comparison purposes, the tensile mechanical properties of the Mg–6Bi alloy extruded by same extrusion parameters from our previous study [22] are also presented in Table 2. Figure 8 shows that the extruded Mg–6Bi–3Zn alloy demonstrates high strength and uniform plastic deformation behavior. The ultimate tensile strength and yield strength reach 336 ± 7.1 MPa and 290 ± 5.5 MPa, respectively. The Mg–6Bi alloy extruded by the same extrusion parameters exhibit 276 ± 2.3 MPa of ultimate tensile strength and 256 ± 5.0 MPa of yield strength [22]. Compared to the strength of the Mg–6Bi alloy, the ultimate tensile strength is increased by 22% for the extruded Mg–6Bi–3Zn alloy. Moreover, as indicated in Table 2, the total elongations of the two alloys are almost the same values, which are 11.5 ± 1.2% and 11.9 ± 0.9%. Therefore, the addition of Zn to the Mg–6Bi alloy can significantly enhance the strength of the Mg–6Bi–3Zn alloy without any loss of ductility.

The experimental results indicate that the microstructure of the extruded Mg–6Bi–3Zn alloy is composed of large unDRXed grains and fine DRXed grains, referred to as a bimodal microstructure. The fine DRXed grains reveal the weakened basal texture due to the occurrence of non-basal textures and a low density of dislocations, whereas a strong intensity of basal texture and high residual dislocation densities exist in large unDRXed grains. It was reported in [20,39,40,41] that the bimodal microstructure in Mg alloys demonstrates high strength and good ductility. Nevertheless, the fine DRXed grains with HAGBs can offer a great contribution to strength by means of grain boundary strengthening. Large unDRXed grains with high dislocation density, as shown in Figure 1b, should provide a great contribution of dislocation strengthening. In addition, the high basal texture intensity in unDRXed grains can provide incremental strength due to the difficult activation of basal slip of dislocation [11,42].

In unDRXed grains with a strong basal texture, the basal slip of dislocations can be suppressed when the sample is drawn along the ED due to the basal plane being parallel to the loading direction. Therefore, considering the difference in microstructure characteristics for fine DRXed grains and unDRXed grains, the modified Hall–Petch equation [43] can be used to understand the contribution of grain size and texture features to the yield strength for bimodal structures, as shown below:

(1) $$∆\sigma_{gb}={f_{DRX}k}_{DRX}{d_{DRX}}^{-1/2}+{f_{unDRX}k}_{unDRX}{d_{unDRX}}^{-1/2}$$

where *d_DRX_* and *d_unDRX_* are the size of DRXed grains and unDRXed grains, which are 3.3 μm and 30.1 μm, respectively. *f_DRX_* and *f_unDRX_* indicate the area fraction of the DRXed grains and unDRXed grains, respectively. They are 75.0% and 25.0%, respectively. The Hall–Petch constants are *k_DRX_* and *k_unDRX_*. In view of the different texture intensities in DRXed grains and unDRXed grains, the *k_DRX_* is 188 MPa μm^1/2^, and *k_unDRX_* is 303 MPa μm^1/2^ [44]. Consequently, the contribution of grain boundary strengthening to yield strength is about 92 MPa.

After extrusion, a large number of residual dislocations is distributed within grains. These residual dislocations are defined as geometrically necessary dislocations (GNDs) [45], which can bring about dislocation strengthening. The dislocation strengthening can be estimated by the following equation [46]:

(2) $${∆\sigma }_{d}=M\alpha Gb\sqrt{\rho^{GND}}$$

In Equation (2), $\rho^{GND}$ is the GND density, which is about 9.4 × 10^14^ m^−2^. *M* indicates the Taylar factor (2.5), α means a constant of 0.2, *b* is the Burgers vector of Mg (0.32 nm) [47] and *G* is shear modulus of Mg (1.66 × 10^4^ MPa) [48]. Therefore, the contribution of dislocation strengthening is calculated to be 81 MPa.

In the present results, about 12.2% content of dynamic precipitates, Mg_3_Bi_2_ phases, with nanoparticle size were obtained. These fine precipitates can result in precipitation strengthening by the Orowan mechanism [49], which is considered another significant factor that significantly improves the strength of extruded Mg–6Bi–3Zn alloys. In general, strengthening by Orowan mechanism can be estimated by the following equation [49,50,51]:

(3) $$∆\sigma_{p}=M\frac{Gb}{2\pi \sqrt{1-\nu }\;\left(\frac{0.953}{\sqrt{f}}-1\right)d_{p}}\ln\frac{d_{p}}{b}$$

where *d_p_* and *f* indicates the average size and area fraction of the Mg_3_Bi_2_ precipitates, which are 39.2 nm and 12.2%, respectively, in the present work. *M* is the Taylar factor (2.5), *b* means the Burgers vector of Mg (0.32 nm), *G* and *ν* indicates the shear modulus (1.66 × 10^4^ MPa for Mg) and Poisson’s ratio (0.33 for Mg) [48], respectively. Therefore, the increment of yield strength by the precipitate strengthening, $∆\sigma_{p}$, is 182 MPa.

Furthermore, the back stress, also defined as hetero-deformation induced (HDI) stress, resulting from hetero-deformation between the hard large unDRXed grains and soft fine DRXed grains, provides an extra strengthening contribution during tensile deformation [20,39,52].

According to the analysis of the Schmid factors (SFs), the SF value of <11$\bar{2}$0> basal slip of dislocations in the extruded Mg–6Bi–3Zn alloy is 0.18, whereas the SF values of non-basal dislocation slip, such as prismatic slip and pyramidal slip, are about 0.45. Therefore, the texture weakening and the easy activation of the non-basal slip of dislocations should be the main reasons for the good ductility of extruded Mg–6Bi–3Zn alloys [31,34,53,54,55].

## 4. Conclusions

This work investigates the influence of Zn addition to the Mg–6Bi alloy on the dynamic recrystallization, texture characteristics, dynamic Mg_3_Bi_2_ precipitates and mechanical properties of the Mg–6Bi–3Zn alloy through hot extrusion at 300 °C. The obtained results indicate that the microstructure of the extruded Mg–6Bi–3Zn alloy exhibits a bimodal structure, which is composed of fine DRXed grains with an average grain size of 3.3 μm and elongated large unDRXed grains along the ED direction with an average size of 30.1 μm. The dynamic recrystallization fraction is 75.0%. It is worth noting that <20$\bar{2}$1>//ED, <44$\bar{8}$3>//ED and <11$\bar{2}$1>//ED non-basal textures are formed in DRXed grains by Zn addition after extrusion at 300 °C, which results in texture weakening for the extruded Mg–6Bi–3Zn alloy. Besides the texture weakening, Zn can promote dynamic precipitation behavior during hot extrusion. Thus, 12.2% of the area fraction of the precipitates and 39.2 nm of the average size of precipitates are obtained. Furthermore, Zn co-existence in the Mg_3_Bi_2_ phase and segregation at grain boundaries are revealed. After extrusion at 300 °C, the Mg–6Bi–3Zn alloy demonstrates an ultimate tensile strength of 336 ± 7.1 MPa and a yield strength of 290 ± 5.5 MPa; meanwhile, the elongation can reach 11.5 ± 1.2%.

## Figures and Tables

**Table 1:** The Taylor rotation axes corresponding to different slip systems in magnesium alloys [29].

Slip System	Slip Type	Taylor Axis
{0001}<11$\bar{2}$0>	Basal <a>	<10$\bar{1}$0>
{10$\bar{1}$0}<11$\bar{2}$0>	Prismatic <a>	<0001>
{10$\bar{1}$1}<11$\bar{2}$0>	Pyramidal I <a>	<10$\bar{1}$2>
{11$\bar{2}$2}<11$\bar{2}$3>	Pyramidal II <c + a>	<10$\bar{1}$0>

**Table 2:** Mechanical properties of the Mg–6Bi and Mg–6Bi–3Zn alloys extruded at 300 °C.

Alloy	*σ*_YS_ (MPa)	*σ*_UTS_ (MPa)	EL (%)
Mg-6Bi [22]	256 ± 5.0	276 ± 2.3	11.9 ± 0.9
Mg-6Bi-3Zn	290 ± 5.5	336 ± 7.1	11.5 ± 1.2

Note: *σ*_UTS_: ultimate tensile strength, *σ*_YS_: tensile yield strength, EL: total elongation.

## Data Availability

The original contributions presented in the study are included in the article, further inquiries can be directed to the corresponding author.
